# Tomography of Laser Powder Bed Fusion Maraging Steel

**DOI:** 10.3390/ma17040891

**Published:** 2024-02-15

**Authors:** Pablo M. Cerezo, Jose A. Aguilera, Antonio Garcia-Gonzalez, Pablo Lopez-Crespo

**Affiliations:** Department of Civil and Materials Engineering, University of Malaga, C/Dr Ortiz Ramos, s/n, 29071 Malaga, Spain; pm@uma.es (P.M.C.); j.a.aguilera@uma.es (J.A.A.); tolin@uma.es (A.G.-G.)

**Keywords:** additive manufacturing, laser powder bed fusion, maraging steel, porosity, X-ray computed tomography

## Abstract

The presence of defects in additive manufactured maraging steel is a widespread problem as its dependence on processing parameters significantly influences it. Using X-ray computed tomography, along with optical microscope data limited to 2D images, quantifies the internal porosity present on a compact tension sample typically employed in fatigue testing. The primary goal of this research is to analyse the pores obtained after the fabrication of a compact tension sample and their main definition parameters, such as sphericity, aspect ratio, surface, and volume, and obtain validation of which method is valid for each of the parameters analysed. The current study aims to enhance the understanding of defects in maraging steel samples through non-destructive 3D analysis. Conventional 2D analyses are limited to surface measurements, providing incomplete information. The proposed method will provide a comprehensive understanding of the defects inside the maraging steel sample, thereby improving the reliability of this material for further applications. This study will contribute to academic and industrial communities by providing a novel approach to analysing maraging steel samples and, ultimately, developing improved materials for various applications. The study’s findings reveal that most pores are produced by gases that are trapped in the fabrication process, and keyhole pores only appear near the surface.

## 1. Introduction

Additive manufacturing (AM) processes have attracted the attention of designers owing to their minimal tooling requirements and the automated layered replication of virtual designs. These processes offer a unique advantage regarding reduced costs and increased flexibility [1,2]. The present method of manufacturing parts has the potential to produce intricate shapes within a significantly shorter time frame than conventional methods. Nevertheless, the manufactured parts may still be subject to quality issues, including porosity and entrapped material that have not been processed.

The field of AM has made remarkable strides and is now recognised as a crucial component in structural engineering. However, the mechanical and fatigue behaviour of newly developed polymers and alloys may still be uncertain [3]. A comprehensive understanding of the fatigue response must be complemented by the use of AM techniques for primary structures. This would aid in enhancing the strength and dependability of material knowledge [4,5], thereby ensuring greater efficiency and effectiveness of the AM process.

Recent research has revealed that the fatigue response of AM technologies is highly sensitive to microstructure [6,7] and defects [8,9], which can significantly influence the bulk properties of fabricated components. The degraded performance of AM structures, compared to structures made using conventional manufacturing techniques, is a matter of concern. Specifically, when AM structures are subjected to ultimate failure via fatigue loading [10,11] or crack growth [12,13], their performance is shown to be lower. Interestingly, the bulk tension or compression testing methods employed during manufacturing are not significantly different from those used in other techniques [14].

The defects and segregation that appear during the printing process have been related to the poorer performance of the pieces fabricated by laser powder bed fusion (LPBF) compared to conventional methods [15,16,17]. Despite the seemingly uniform nature of laser-based manufacturing processes, micrographs reveal the irregular growth of fine grains and the remaining boundaries of different melt pools [18,19]. These factors can impact the fatigue of the material. In constructing a larger structure with intricate details [20,21,22,23], it is essential to understand the flawed characteristics of the material to avoid unexpected failures [24].

Maraging steel alloys are widely employed in various industries, including the aerospace and aircraft sectors, tool steel applications, or automotive components, such as crankshafts and gears. Another example of its use is in the fabrication of foil and épée fencing blades because maraging steel has a much lower crack growth rate than carbon steel, which in turn means higher resistance against the fracture of the blade and consequently increased safety for the practice of the sport. In addition, it is used in bicycle frames, golf clubs, and surgical components and hypodermic syringes. The reason for such widespread use is their exceptional mechanical properties, high hardness, and dimensional stability. These alloys are renowned for their superior mechanical strength, good weldability, and machinability, and they are thus considered a prime choice for industrial applications that demand uncompromising performance and reliability. In summary, maraging steel alloys are used for applications that demand high hardness, high mechanical strength, and dimensional stability [25,26,27,28,29].

The uncertain nature of the fatigue performance of AM technologies [30] must be mitigated because they are generally part of high-value-added mechanisms, and their failure would result in large economic losses. Local porosity, modulus, microstructure phases, and hardness measurements have been demonstrated as reliable indicators of failure nucleation, fatigue damage, and failure in non-AM techniques [31,32,33,34]. Experimental techniques like X-ray computed tomography (XCT), atomic force microscopy, and nanoindentation [35,36] can be used to evaluate very small quantities of material, even between layers in AM, and they can provide accurate knowledge of how the failure mechanism works [37].

XCT is a highly effective, non-destructive inspection technique used to assess various materials’ physical and geometrical characteristics. It is beneficial for evaluating internal structures and features of materials like metals, ceramics, plastics, polymers, mixed composites, components, assemblies, etc. One of the most significant advantages of using XCT is that it generates densitometric images of thin cross-sections through an object without causing any damage to the material [24,38].

While XCT is a fantastic non-destructive evaluation method for inspecting complex and dense materials, obtaining accurate scan results can be challenging when dealing with large and thick objects, particularly those made of steel or other highly attenuating materials. This can lead to low-intensity throughput results, low image contrast, low signal–noise ratio, and image artefacts.

AM processes can produce three distinct types of pores: keyhole pores, lack of fusion pores, and gas pores [39]. Keyhole pores form when excessive energy is applied during the melting process, causing the metal powder to penetrate deeply and form a pore near the bottom of the solidified melt pool. These pores typically have a circular shape in the horizontal plane and an elongated shape in the vertical plane [40]. Lack of fusion pores occur when insufficient energy is supplied to the powder bed during melting, causing the metal powder to fail to melt entirely and creating voids in the final structure. These pores are irregular and large, comparable to the melt pool size [41]. Finally, gas pores are the smallest and most spherical of all, and they are formed by trapped gases that are either present in the metal powder or introduced during melting [42,43].

Although LPBF technology can achieve high-density rates of approximately 99%, the residual porosity in LPBF-fabricated parts presents a significant challenge for their practical utilisation in high-strength and fatigue-resistant applications [44]. Components made using LPBF have mechanical properties that are affected by their microstructure in the same way as conventionally manufactured parts. Investigating the distribution of microvoids in a structural formation is a critical factor in determining the mechanical properties of the final product. This analysis is vital to optimising the final product and broadening the material’s possible industrial applications.

A comprehensive study was conducted on a sample of maraging steel produced through LPBF. The study employed a meticulous 3D XCT to examine the microvoids present, including their distribution and types. The parameters used to define the pores were then compared to those obtained through a 2D optical microscope (OM) examination.

## 2. Material and Methods

### 2.1. Based Material

The LPBF process uses steel powder particles with a 40 µm diameter obtained from maraging steel 18Ni300 with a well-defined chemical composition, as shown in Table 1. Maraging steel was selected for its remarkable mechanical properties and significance in challenging industrial applications.

### 2.2. Laser Powder Bed Fusion

The experiment used the LPBF technique to produce compact tension (CT) specimens. Steel powder particles were fused using a high-powered laser via Lasercusing^®^ in a layer-by-layer production process. The LPBF process was carried out using the “Renishaw” (Wotton-under-Edge, UK) equipment model “AM 400,” and the research team optimised the process parameters to attain the desired outcomes. The setup encompassed a laser power of 400 W, a scan speed of 0.85 m/s, and a laser diameter of 0.04 mm. The thickness of each deposited layer was 30 µm, and a hatch spacing of 100 μm with 25% overlapping was utilised.

**Figure 1:**
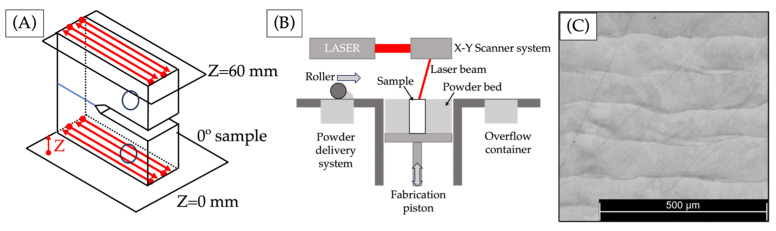
(**A**) LPBF CT specimen orientation pattern (red lines are weld beads’ orientation, and the blue line is the crack plane), (**B**) diagram of LPBF process, and (**C**) welding beads of the additive manufacturing maraging steel sample.

Figure 1A displays the deposition pattern of the CT specimen for a 0° orientation. Each layer has been deposited in parallel with the presumed pathway for the crack to propagate. The angle corresponds to the crack’s direction of propagation, and the pattern was followed from z = 0 to z = 60 mm after the notch and holes were machined. Figure 1B depicts a schematic representation of the laser powder bed fusion (LPBF) printing process, which involves the deposition of successive layers of welding beads. Following a polishing and etching step, Figure 1C shows the final structure of the sample, which is characterized by the presence of the aforementioned sequence of welding beads that have been deposited layer by layer.

### 2.3. Defects Characterisation

Previous research has found pores of 5–10 µm in diameter on 2D sections [45], so in order to ensure accurate characterisation testing, it is necessary to reduce the resolution of the sample enough to account for the existence of pores. A small parallelepiped from the overall specimen was extracted from the surface of the specimen. This was carried out because of the high absorptivity of the material towards the X-rays, increasing the thickness of the sample. Increased power is needed, and a lower resolution is acquired.

**Figure 2:**
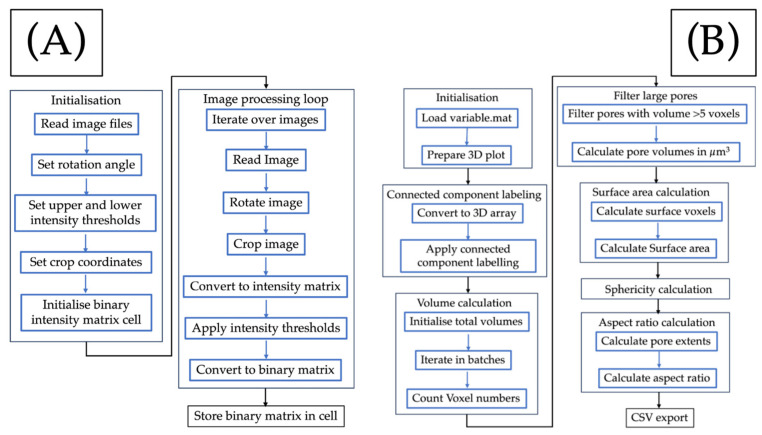
Flow charts of (**A**) pore thresholding obtention and (**B**) reconstruction of 3D pores through the separated slides provided after X-ray CT.

X-ray computed tomography (CT) is performed using SkyScan 2214 CMOS edition by Bruker (Madrid, Spain) and a voxel size of 1.5 µm. The sample underwent scanning with 150 kV voltage and 90 µA intensity. The process involved 1350 projections and took approximately 6.5 h. To reconstruct the 3D image, MATLAB R2022b software was used, and Python 3.11 software, with the Library Scipy, was utilised for data analysis. Manual thresholding was performed using the algorithm in Figure 2A, and analysis was conducted according to Figure 2B. Pores smaller than 5 voxels were removed. The smallest detectable pore equivalent diameter was 4.5 µm, meaning the resolution of defects is 4.5 µm in diameter.

**Figure 3:**
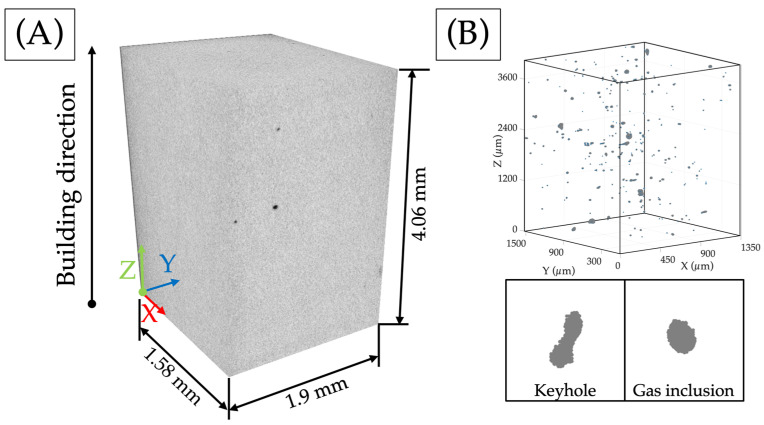
(**A**) CT sample section after X-ray tomography was reconstructed in FIJI; (**B**) porosity in the volume analysed and main porosity types.

The reconstructed 3D samples were used to examine internal defects, as shown in Figure 3B. To determine the relative density of the parts, the ratio between the volume of the sample (without pores) and the total volume of pores was computed from 3D images via the Python routine shown in Figure 2B. Figure 3A displays a sample that measures 4.06 × 1.9 × 1.58 mm, along with the direction in which it was constructed. An image of the scan is presented in FIJI, revealing the presence of pores on the surface of the analysed sample. Figure 3B provides a closer look at the pores within the sample, identifying two primary types of porosity: elongated keyhole pores with a significant volume and spherical gas inclusion pores with a smaller volume.

For each pore, the aspect ratio and sphericity were calculated apart from measuring the volume and the surface area. Sphericity is calculated for a 3D object by knowing the surface area and volume. According to Wadell [46], the sphericity of an object can be defined as the ratio of the nominal surface area (which is the surface area of a sphere that has the same volume as the object) to the actual surface area of the object. This ratio is known as the true sphericity index, as shown in Equation (1).

(1) $$f_{SPH}=\frac{A_{n}}{A}=\frac{{\left(36\pi V^{2}\right)}^{\frac{1}{3}}}{A}$$

Variables *V*, *A_n_*, and *A* are the volume, nominal surface area, and surface area, respectively. The sphericity index, a measure of how closely an object approaches a sphere in its geometry, attains a value of 1 for a perfect sphere. Isoperimetric inequality asserts that any object that deviates from this ideal geometry will possess a sphericity value that is strictly less than 1.

The sample was also examined in an OM, specifically the Leica DM6 M (Wetzlar, Germany), to observe three cross-sections. Previously, the cuts were mechanically polished until one-micro diamond powders were obtained to attain a mirror-like finish. The size of the pores was determined using a pixel-counting method with the help of commercial software called FIJI. Additionally, each pore was assessed based on its circularity to determine its proximity to a circular cross-section using Equation (2).

(2) $$f_{circ}=\frac{4\pi A}{p^{2}}$$

Variables *p* and *A* represent the perimeter and the cross-sectional area of the pore, respectively. Circularity factor $f_{circ}$ takes values within interval $0<f_{circ}\leq 1$, with the value of 1 indicating a circular cross-section [47].

The aspect ratio is provided in Equation (3).

(3) $$f_{aspect}=\frac{d_{min}}{d_{max}}$$

Parameters $d_{max}\;\mathrm{and}\;d_{min}$ are the pore’s maximum and minimum orthogonal diagonal. The aspect ratio $f_{aspect}$ takes values in the interval $0<f_{aspect}\leq 1,$ where any other value than 1 implies that the pore is elongated. The shape factor is determined by the contour’s regularity and the pore’s shape.

Due to the restrictions of the testing apparatus and the high absorptivity of the material, the attained resolution is approximately 1.5 µm. This consequently gives rise to a significant margin of error, particularly when measuring minute pore volumes. However, as the pore volume increases, this error is mitigated.

## 3. Results and Discussion

**Figure 4:**
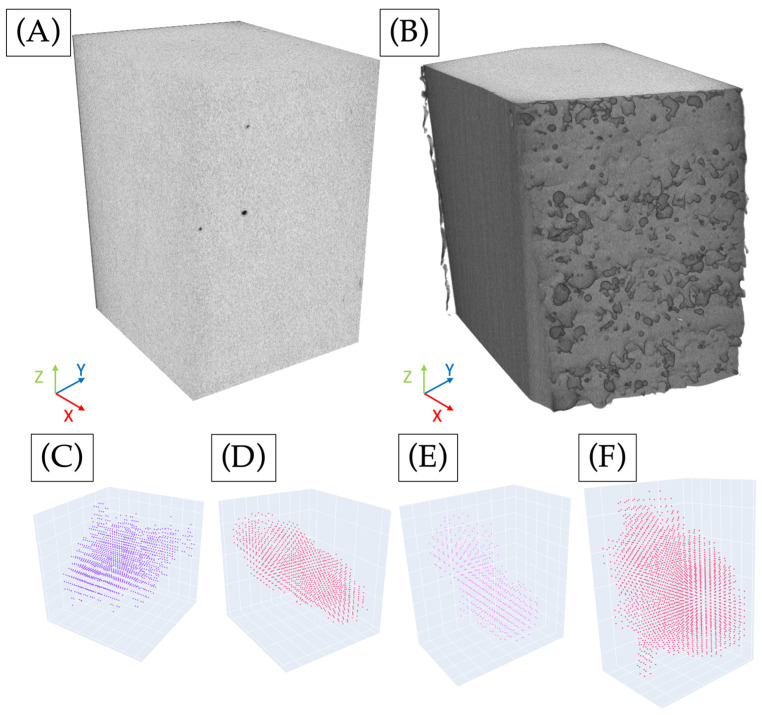
Sample X-ray CT output cross-section of a parallelepiped core. (**A**) Segmented image, (**B**) original image, and examples of pore volumes: (**C**) 1474 µm^3^, (**D**) 1236 µm^3^, (**E**) 583 µm^3^, and (**F**) 2285 µm^3^.

LPBF-manufactured samples have an inherent porosity that negatively affects the mechanical behaviour of fabricated samples. The present study involved the analysis of a specific portion of the sample, as shown in Figure 4B, through the utilisation of X-ray computed tomography (CT) imaging and subsequent image processing, as previously alluded to. 

Figure 4A displays the original sample’s internal image and the entire portion of the sample that was scrutinised for analysis. The boundary of the specimen is explicitly demarcated, and the internal defects are identified by a change in colour, appearing as black or darker grey. The surface that is fabricated last can be identified when the value of X reaches its maximum.

**Figure 5:**
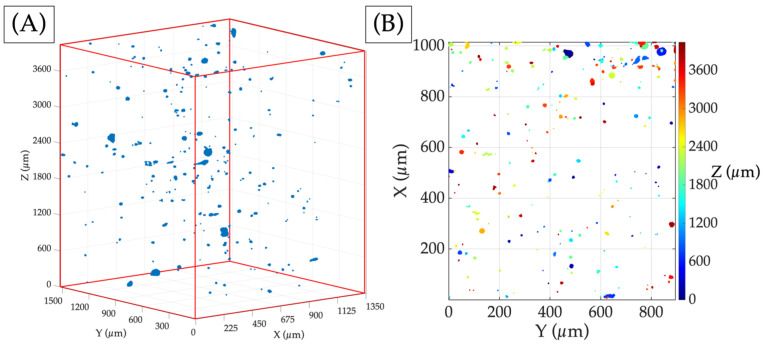
(**A**) Pores presented in the sample volume analysed, where blue dots show the pores and red lines indicate the specimen’s boundaries; (**B**) porosity projection of the hole’s volume on the X-Y plane, where the colour bar represents the height of each pore.

The images provided by X-ray tomography are segmented, and after the treatment specified in the Section 2, the porosity detected in the scanned volume is represented in Figure 5A, where blue indicates the voids throughout the parallelepiped and the red lines indicate the specimen’s boundaries. Initially, the voids are observed to be spread evenly throughout the volume, as they have been followed before [24], but when the projection of the porosity is represented in Figure 5B, it is clearly shown that there are concentrations of the pores close to the surface of the sample when the X and Y axes are more significant, as shown in Figure 4B. Such pores are less visible in Figure 5A due to their position near the surface, which blends with the white background.

There may be a correlation between the appearance of pores in the printed sample and the laser beam’s acceleration and deceleration at the sample’s edges. This can lead to higher energy densities that result in the formation of pores. Alternatively, the weld beads at the beginning and end of each cylindrical layer may be shorter than those in the central part of the layer. This can cause the beads to have less time to cool between each laser run, potentially contributing to pore formation. It is worth noting that these pores are only visible near the surface of the sample since each layer is printed at a 0° angle with respect to the preceding layer [48].

The presence of internal defects in powders can be attributed to two distinct types of pores—spherical pores resulting from the formation of gas inclusions (complete spherical shapes) or keyholes (semispherical shapes, caused by the union of partial spheres), as shown in Figure 3B, and non-spherical pores arising from a lack of fusion.

In cases with excessive laser energy input, slow scan velocity, and high power, the metal surface experiences vaporisation, leading to recoil pressure that pushes down on the melt pool surface. This pressure causes the creation of a narrow and deep keyhole, where various laser reflection and absorption events occur [49,50]. The laser’s uneven absorption on the surface can lead to localised hotspots, causing an imbalance between capillary force, vapour dynamic pressure, and recoil pressure. In unstable keyhole conditions, it has been observed that gas bubbles tend to pinch off at the keyhole’s tip [51,52,53,54].

According to previous research [45], the distribution of the atomic composition of pores does not result in an uneven precipitation of alloy elements. In this research, all elements maintained a stable distribution with a slight reduction in the case of Fe and an increase in Ni when moving away from the centre of the pore.

**Figure 6:**
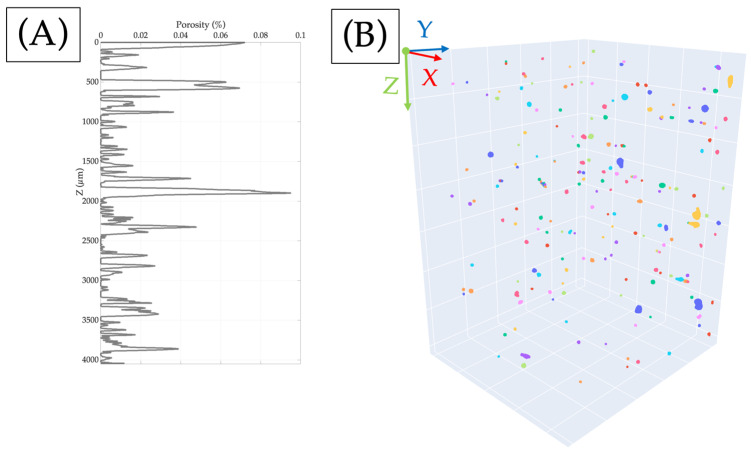
(**A**) The percentage of porosity of the sample obtained through X-ray CT and (**B**) segmented image with the coordinate axis used in the previous image and pores are shown in a different colour.

The previous literature has shown that through the LPBF method, high densities can be obtained [48,55]. Relative densities are more elevated than 99.7% for the samples studied before [48], and the relative porosity does not vary along the construction axis. The porosity profile of the 18Ni300 sample along construction axis Z is illustrated in Figure 6A. The results indicate that the relative porosity is less than 0.1%, which implies that the relative density exceeds 99.9% for all sections analysed, even when accounting for the surface porosity. The porosity obtained through OM can reach up to 0.185% of the section.

**Figure 7:**
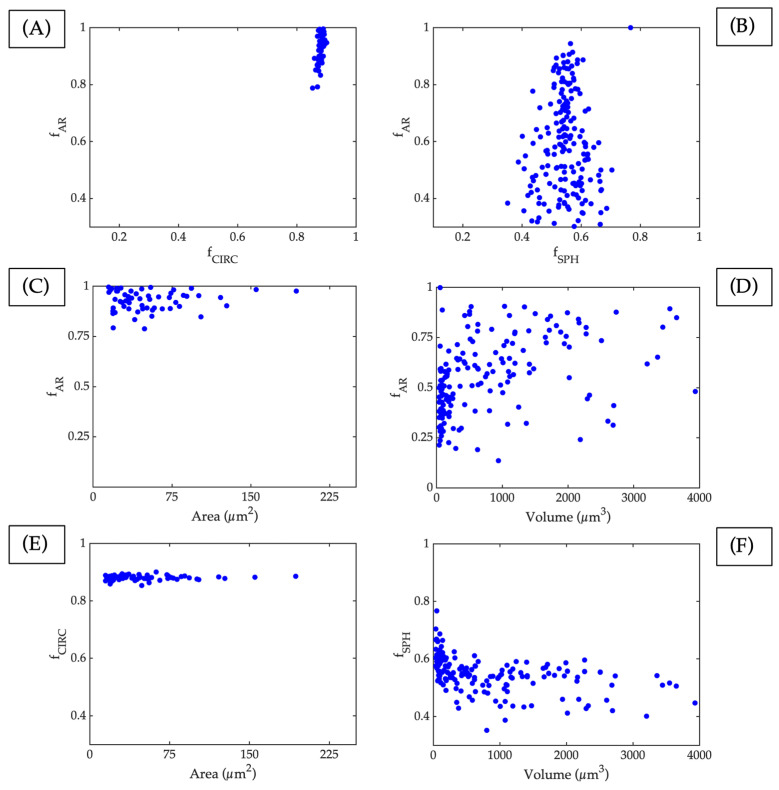
Relation between main definition parameters of pores measured through X-Ray CT (3D) or OM (2D), each blue circle is a different pore. (**A**) Aspect ratio vs. circularity in 2D, (**B**) aspect ratio vs. sphericity in 3D, (**C**) aspect ratio vs. area in 2D slides, (**D**) aspect ratio vs. volume in 3D, (**E**) circularity vs. area in 2D slides, and (**F**) sphericity vs. volume in 3D.

Upon the completion of CT measurements, a comparative analysis was conducted with surface porosity research performed via OM. This approach facilitates the evaluation of variances between the 2D and 3D methods of porosity characterisation. The analysis depicted in Figure 7 illustrates the relationship between values obtained in two dimensions, such as the circularity factor and pore area, with their respective values in three dimensions, such as the sphericity factor and volume. Additionally, the aspect ratio has a direct correlation.

Figure 7A,B visually represent the correlation between the aspect ratio and circularity in 2D and 3D, respectively. The outcomes of the study highlight a significant difference between both methods, with the 2D values being higher for circularity and the aspect ratio, typical of pores that are caused by trapped gas. Conversely, the results obtained in 3D are centred on average sphericity values. The variation observed in this parameter could potentially be attributed to the fact that 2D cutting often yields a circular shape when one of the pore planes is cut. As a result, most pores are expected to have a more uniform shape, while the third unmeasured measurement (supplemented by 3D analysis) accounts for pore non-uniformity.

A previous research study [43] defined this region as the “unclear region”; however, it can now be defined within the keyhole. The largest dimension is taken by definition among the smallest, meaning the aspect ratio value will not be higher than 1 in any of the cases.

Figure 7C,D plot the aspect ratio against the area (2D) and volume (3D), respectively. The results demonstrate that the aspect ratio is more widely distributed in 3D, but the pore size distribution is similar to that obtained in 2D. This is because a more extensive analysis is carried out in 3D, which allows for more precise maximum and minimum pore size values than those obtained only on a surface in 2D by means of an OM. Figure 7D,F show a concentration of pores below 500 µm^3^ that can be addressed relative to gas-trapped pores with corresponding higher sphericity values according to [43].

Lastly, Figure 7E,F explore the relationship between circularity (2D) and sphericity (3D) with respect to area (2D) or volume (3D), respectively. The results show that sphericity presents values well below those offered by circularity, and this is possibly due to the abovementioned reasons. However, pores with low volumes show higher values of sphericity than those with higher volumes. This could be a symptom that they can be classified into pores created by gas trapped during their manufacture. Sphericity decreases as the pore’s volume increases, and this is possibly due to the “keyhole effect”.

Based on the measurements taken, it appears that there is a higher concentration and larger volumes of pores on the surface area of the piece, which may be due to the formation of “keyhole pores” resulting from an excess of energy. These pores are located close to the surface and do not undergo further deposition or re-melting. Re-melting would mean that the energy applied is less than that needed to create a keyhole pore, making them disappear when analysing deeper slides on the samples. This phenomenon has been observed before [56], indicating that pores with high volumes and moderate sphericity values may be caused by the “keyhole effect”.

The shape of keyhole pores creates stress concentration areas that weaken the material’s mechanical strength, making it susceptible to cracks during fatigue and less reliable. The unpredictability of where these pores may appear leads to a decrease in material reliability as their number increases. However, smaller and less damaging pores caused by trapped gas can also impact the material’s reliability. However, they have a smaller impact on crack initiation and overall reliability compared to keyhole pores.

It is important to note that machine testing has been restricted to a limited section of the surface sample as a result of the material’s high level of absorptivity. As a result, additional research is needed to enhance the strength and reliability of maraging steel printed through LPBF and expand the study’s scope.

## 4. Conclusions

In conclusion, the current study has provided valuable insights into the microstructure and microhardness of 18Ni300 fabricated via LPBF. The study presents valuable information on the porosity generated after the additive manufacturing of maraging steel, in which the pores are identified via X-ray CT and compared with the pores analysed via optical microscopy techniques, thus identifying a larger number of pores and more correct and accurate pore values. The results of this study have significant implications for future research in this area and are expected to contribute to the development of more efficient manufacturing processes for 18Ni300 and similar materials. After conducting a thorough investigation, the research has led to the following conclusions:

(1) A greater occurrence of pores is observed in the regions adjacent to the surface where the final layer of material is applied. Due to their expansive surface area, these pores may be classified as keyhole pores. Notably, the values are not represented in 2D, as only a single slice is evaluated in each study. Moreover, since the pore shape is probably spherical, it is plausible that the obtained value may differ from the actual value. Thus, OM is not recommended for studying the type of pore in the surroundings of the surface.
(2) Keyhole pores only appear in surface slides with the used printing configuration as the deposition of another layer on top of the last one makes them disappear after reheating.
(3) Based on the stability of density above 99.9%, which is similar to the results obtained by OM, it can be concluded that both methods are valid and accurate.

## Figures and Tables

**Table 1:** Chemical composition of 18Ni300 maraging steel.

[wt/%]	Fe	Ni	Co	Mo	Ti	Cr	Si	Al	Mn	C	P
18Ni300	Balance	18.2	9.0	5.0	0.6	0.3	0.1	0.05	0.04	0.01	0.01

## Data Availability

Data are contained within the article.
